# Prediction of Overall *In Vitro* Microsomal Stability of Drug Candidates Based on Molecular Modeling and Support Vector Machines. Case Study of Novel Arylpiperazines Derivatives

**DOI:** 10.1371/journal.pone.0122772

**Published:** 2015-03-31

**Authors:** Szymon Ulenberg, Mariusz Belka, Marek Król, Franciszek Herold, Weronika Hewelt-Belka, Agata Kot-Wasik, Tomasz Bączek

**Affiliations:** 1 Department of Pharmaceutical Chemistry, Faculty of Pharmacy, Medical University of Gdańsk, Gdańsk, Poland; 2 Department of Drug Technology and Pharmaceutical Biotechnology, Medical University of Warsaw, Warsaw, Poland; 3 Department of Analytical Chemistry, Chemical Faculty, Gdańsk University of Technology, Poland; National Research Council of Italy, ITALY

## Abstract

Other than efficacy of interaction with the molecular target, metabolic stability is the primary factor responsible for the failure or success of a compound in the drug development pipeline. The ideal drug candidate should be stable enough to reach its therapeutic site of action. Despite many recent excellent achievements in the field of computational methods supporting drug metabolism studies, a well-recognized procedure to model and predict metabolic stability quantitatively is still lacking. This study proposes a workflow for developing quantitative metabolic stability-structure relationships, taking a set of 30 arylpiperazine derivatives as an example. The metabolic stability of the compounds was assessed in *in vitro* incubations in the presence of human liver microsomes and NADPH and subsequently quantified by liquid chromatography-mass spectrometry (LC-MS). Density functional theory (DFT) calculations were used to obtain 30 models of the molecules, and Dragon software served as a source of structure-based molecular descriptors. For modeling structure-metabolic stability relationships, Support Vector Machines (SVM), a non-linear machine learning technique, were found to be more effective than a regression technique, based on the validation parameters obtained. Moreover, for the first time, general sites of metabolism for arylpiperazines bearing the 4-aryl-2H-pyrido[1,2-c]pyrimidine-1,3-dione system were defined by analysis of Q-TOF-MS/MS spectra. The results indicated that the application of one of the most advanced chemometric techniques combined with a simple and quick *in vitro* procedure and LC-MS analysis provides a novel and valuable tool for predicting metabolic half-life values. Given the reduced time and simplicity of analysis, together with the accuracy of the predictions obtained, this is a valid approach for predicting metabolic stability using structural data. The approach presented provides a novel, comprehensive and reliable tool for investigating metabolic stability, factors that affect it, and the proposed structures of metabolites at the same time. The performance of the DFT-SVM-based approach provides an opportunity to implement it in a standard drug development pipeline.

## Introduction

Metabolic stability is a factor that often excludes potential drug candidates from any further studies. In 2002, the average development time for a new chemical entity (NCE) up to registration was 12 years. In the same year, according to the US Food and Drug Administration, only 17 new drug candidates were approved, which represents the lowest approval rate in the past decade. Other studies show that in 1991, the major reasons for the failure of NCEs to obtain regulatory approval were inadequate metabolic and pharmacokinetic (PK) parameters [1]. This has lead to the development of new approaches dedicated to increasing the rate of NCE approval. One of these is an *in vitro* approach for early determination and prediction of the metabolism of a drug [2]. Of the many advantages this approach provides, one of the greatest is determination of a metabolic profile for the NCE earlier in the drug development process. Conclusions drawn from this research stage can be used to design chemical modifications of the next generation of NCEs, which can further improve their metabolic stability. Secondly, *in vitro* studies allow for utilization of human enzymes, liver fractions or cells, thus providing data that reflects the *in vivo* situation without sacrificing a large number of animals and, importantly, minimizing costs.

Both the prevalence and variety of mood disorders have induced the development of new drugs for their treatment. Selective serotonin reuptake inhibitors (SSRIs) are one of the most popular groups of drugs for treating depression. One of their major flaws, however, is a long onset period for clinical effect, which can take as long as 3–4 weeks. Starr and coworkers stated a hypothesis to explain such a mechanism, based on a competitive blockade of 5-HT_1A_ auto-receptors and serotonin reuptake inhibition [3]. It has been reported that administration of paroxetine, a 5-HT_1A_ partial antagonist, together with pindolol, a β-adrenergic receptor antagonist, can reduce the onset time of anti-depressive action from 2–3 weeks to 3–7 days [4]. When considering other 5-HT receptor ligands, arylpiperazine derivatives are a confirmed group of substances that have mostly 5-HT_1a_ and/or 5-HT_2a_ affinity. As a relatively new class of psychotherapeutic drugs and drug candidates, they are partial agonists, showing high affinity towards a 5-HT_1A_ receptor binding site, yet low selectivity for this receptor subtype. A model drug from this group, buspirone, along with its derivatives trazodone and etoperidone, has gained wide acceptance for treating psychotic disorders accompanied by anxiety. All of the abovementioned properties make arylpiperazines worthy candidates to combine with SSRIs, not only to decrease the time of onset for clinical anti-depressive effects, but also to help patients withstand the hardships of therapy and related stressors.

Among recently developed novel arylpiperazine derivatives, structure-activity relationship [5–10] and molecular modelling of target receptor studies [11] have been quite popular. Unfortunately, relationship between structure and metabolic stability hasn’t been so thoroughly studied. Hence, it is necessary to develop a protocol, including biotransformation, analytical quantification and a reliable algorithm, to describe and predict metabolic stability within this group.

Support Vector Machines is a technique that has been broadly implemented in metabolism studies. Applications for this method range from predicting possible sites of metabolism to predicting substrates for CYP isoforms and UGT-interacting molecules, using SVM classification [12–15]. To the best of the authors’ knowledge, SVM has not been used to predict half-life values as a measure of metabolic stability.

The aim of this study was to propose and validate a comprehensive workflow to study metabolic stability with 30 model arylpiperazine derivatives. The products of *in vitro* biotransformations utilizing microsomes were analyzed by liquid chromatography-tandem mass spectrometry (LC-MS). The obtained experimental results are supported by density functional theory (DFT) calculations and model building based on the support vector machine (SVM) technique. Quadrupole-time-of-flight (Q-TOF) MS/MS fragmentation spectra provide complementary information about a complex situation of arylpiperazine metabolic reactions.

## Materials and Methods

### Chemicals

Materials used for incubations included: pooled human liver microsomes and sodium salt of NADPH (Sigma-Aldrich, Saint-Louis, MO, USA), monopotassium phosphate and dipotassium phosphate (POCH, Gliwice, Poland), and deionized water, 18 MΩ resistance (Millipore, Bellerica, MA, USA). Dimethyl sulfoxide (Sigma-Aldrich) was also used to prepare stock solutions of compounds 5 and 6. The LC-MS internal standard was a 5 μM buspirone hydrochloride solution (Sigma-Aldrich). The mobile phase consisted of deionized water, HyperGrade LC-MS acetonitrile (MERCK, Darmstadt, Germany) and formic acid for LC-MS (Sigma-Aldrich).

### Microsomal incubation and sample preparation for LC-MS

The studied arylpiperazines were synthesized in the Department of Drug Technology and Pharmaceutical Biotechnology at the Medical University of Warsaw as described earlier (Herold et al., 2006). The studied compounds were dissolved in acetonitrile (compounds 1, 3, 4, 10, 18, 19, 21, 22, 23, 24, 25, 26, 27, 28, 29), water (compounds 2, 7, 9, 11, 12, 13, 14, 15, 16, 17, 20, 30) or dimethyl sulfoxide (compounds 5 and 6), and diluted in phosphate buffer (pH = 7.4) to obtain 100 μM solutions. A 10 mM NADPH solution in phosphate buffer was prepared before incubations. Final concentrations in the incubation mixes were 5 μM of studied compound, 500 μM of NADPH and 1 mg/ml of human liver microsomes.

Using a MyBlock mini dry-bath (Benchmark Scientific, NJ, USA), mixtures without the compounds were subjected to a 5 min pre-incubation at 37°C before each experiment. Incubations were initiated with the addition of 15 μL of the compound solution to the mixture. The experiment lasted for 30 min. Immediately after addition of the compound, 50 μL of incubation mixture was collected and mixed with 50 μL of the stopping reagent, which was ice-cold acetonitrile containing 5 μM buspirone as internal standard. The next sample collection points were after 5, 10, 15 and 30 minutes of incubation time. After stopping the reaction, suspensions were centrifuged for 10 minutes at 7378 g using a Microfuge 16 (Beckman Instruments, Fullerton, CA, USA). Subsequently, 70 μL of supernatant thus obtained was directly analyzed using LC-MS (Agilent Technologies, Santa Clara, CA, USA).

The incubation and LC-MS analysis were repeated twice for each compound. To ensure that the biological conditions were valid, and no compound degradation occurs related to the addition of the enzyme solution, a negative control for each derivative was also performed. The composition of negative control mixtures was similar to that used in experiment, with the only difference being the absence of NADPH. Without cofactor, microsomes are deprived of their normal activity, and therefore, after performing incubation and LC-MS analysis, the concentration of the studied compound should not be significantly altered.

### LC-MS assay for t_0.5_ determination

The LC analysis was carried out using an Agilent 1260 Infinity system (Agilent Technologies) consisting of a binary pump, autosampler, temperature-controlled column compartment and UV variable wavelength detector, coupled with an Agilent 6120 Single Quad mass detector. Incubation samples were separated at 40°C on a Poroshell 120 EC-C-18 column (3 mm x 100 mm, 2.7 μm), provided by Agilent Technologies. The data were collected and processed in ChemStation software (v. B. 04.03, Agilent Technologies, Santa Clara, CA, USA). The injection volume was 10 μL. Mobile phases consisted of deionized water (phase A) and acetonitrile (phase B), both with 10 mM ammonium formate buffer (pH = 3) added. Gradient elution program was as follows: 0–15 min—5%-100% B, 15–16 min—100% B, 16–16.10 min—100%-5% B, 16.10–21 min 5% B. The flow rate was 0.5 ml/min. The total time for a single analysis was 21 minutes. MS detection was performed using an Agilent 6120 Single Quad mass spectrometer (Agilent Technologies) equipped with an electrospray (ESI) ion source. A nebulizer (pressure set to 40 psig) allowed for the gas phase ionization of studied compounds. Nitrogen (NM32LA Nitrogen Generator, Peak Scientific Instruments, Billerica, MA, USA) was used as the drying gas, with a flow rate of 10 L/min and a drying temperature of 300°C. Capillary voltage was set to 3000 V. Fragmentor voltage was set to 150 V. Chromatograms were collected with the use of single ion monitoring (SIM) in positive ion mode. Mass to charge ratios (m/z) for all compounds are gathered in Table 1.

**Table 1:** Compound moieties, pseudo-molecular ion masses and values for estimated half-life.

**Compound number**	**R**	**R'**	**R”**	**[M+H]** ^+^ **[m/z]**	**Average T**_1/2_ **[min]**
1	CH_3_	H	3-CF_3_-Ph	537.24	4.51
2	CH_3_	H	2-pyridyl	470.25	4.57
3	CH_3_	H	2-Cl-Ph	503.22	4.12
4	CH_3_	H	2-F-Ph	487.25	5.52
5	CH_3_	H	2-pyrimidinyl	471.25	3.52
6	H	H	2-pyrimidinyl	457.23	5.29
7	H	H	2-pyridyl	456.23	6.02
8	H	H	3-CF_3_-Ph	523.23	5.40
9	OCH_3_	H	2-pyridyl	486.24	4.44
10	OCH_3_	H	2-F-Ph	503.24	4.87
11	OCH_3_	H	2-pyrimidinyl	487.24	4.00
12	Cl	H	2-pyridyl	490.2	3.31
13	Cl	H	2-pyrimidinyl	491.12	2.76
14	F	H	2-pyridyl	474.22	4.41
15	F	H	2-pyrimidinyl	475.22	4.40
16	H	F	2-pyridyl	474.22	7.04
17	H	F	2-pyrimidinyl	475.22	9.25
18	H	H	2-Cl-Ph	493.23	5.49
19	H	OCH_3_	2-F-Ph	507.27	6.57
20	H	OCH_3_	2-pyridyl	490.28	6.83
21	H	OCH_3_	2-pyrimidinyl	491.27	7.42
22	Cl	H	3-CF_3_-Ph	561.22	4.40
23	CH_3_	H	2-pyrimidinyl	475.28	5.24
24	OCH_3_	H	2-Cl-Ph	523.24	3.26
25	H	H	2-F-Ph	479.28	5.77
26	F	H	2-Cl-Ph	511.22	4.21
27	F	H	3-CF_3_-Ph	545.25	4.14
28	F	H	2-CH_3_-Ph	491.28	3.77
29	F	H	2-pyridyl	478.26	6.81
30	H	H	2-pyrimidinyl	461.26	9.32
Buspirone					6.59

### Half-life evaluation of the studied compounds

Half-life evaluation requires a compound depletion plot, which is obtained by plotting the time of incubation against the logarithmic peak area ratio (the ratio of the compound peak area to the internal standard peak area). To calculate the half-life in the present study, only the linear part of a compound’s depletion plot was used. To calculate the gradient, the *a* constant (or the slope of the function) from the linear function equation was used. After the elimination rate constant was designated, half-lives were calculated (Equation 1).

$$\begin{array}{c} Elimination\;rate\;constant\left(k\right)=-gradient\\ Halflife\left(t_{0.5}\right)\left(min\right)=\frac{0.693}{k} \end{array}$$ Equation 1 Half-life evaluation equation.

### Numerical representation of the compounds’ optimized 3D structures

Three-dimensional structures of the studied compounds were assessed using an *in silico* approach. Two-dimensional models of the studied compounds were created manually in ACD ChemSketch (Advanced Chemistry Development, Inc., Toronto, Canada) and thereafter optimized. A semi-empirical AM1 force field was applied using HyperChem (v 8.0.8, HyperCube, Gainesville, FL, USA) to obtain a pre-optimized model, which was subsequently subjected to density functional theory (DFT) calculations. The AM1 force field pre-optimization was performed in order to shorten the DFT calculation portion of the optimization process. DFT calculations were conducted using Gaussian software [16] at the B3LYP/6-311 G(d) level of theory.

A set of 4870 descriptors was calculated from the optimized structures with the use of DRAGON 6.0 Software (Talete, Milano, Italy). After excluding descriptors that had constant values, 2787 descriptors (divided in blocks, such as edge adjacency indices, 2D autocorrelation and geometrical descriptors) were imported into STATISTICA 10.0 software (Statsoft, Tulsa, OK, USA) to perform further statistic and chemometric calculations.

### Statistic and chemometric approaches

Statistical analysis was performed using STATISTICA software. The dataset, comprising 30 compounds, was divided into two datasets—a training set containing 22 compounds, and a test set comprising 8 compounds, chosen randomly. All of the half-life values were transformed using a y = log(x) function to provide a normal distribution in accordance with the Shapiro-Wilk test (p>0.05). The data matrix was also standardized. Using the data mining and feature selection functions of Statistica software, the 6 most relevant descriptors were chosen from the whole training dataset. Feature selection is a technique used to develop a subset of the most relevant features. A theory behind this technique is that large datasets contain features that are irrelevant or redundant. Feature selection excludes them and limits the dataset to only the most relevant features, simplifying the model that is created afterwards and making it easier to interpret.

The support vector machines (SVM) method is a model that has been developed by Vapnik [17], and the theory behind support vector regression has been extensively described [18]. The basic concept behind SVM is to map the studied data into a higher dimensional feature space using a kernel function, K (*x*, *x*_*i*_). In SVM classification, a model is a representation of points mapped in space, divided by a gap that separates classes from each other. In support vector regression, model represents points mapped in space in a way that allows to create a regression line. A regression is then performed, providing a model for predicting values. In the present study, the optimization option of the winSVM [19] program was used to determine values for the method parameters (capacity and epsilon value) and kernel type, which were used later to perform SVM in STATISTICA software. After 100 optimization runs with the training set, a combination of parameters producing the lowest mean square error was chosen. In this case, a type 1 SVM regression was used (capacity = 10, epsilon = 0.1). The radial basis function was used as a kernel, with gamma = 0.9.

### LC-Q-TOF-MS/MS assay for metabolites structure elucidation

Tandem mass spectra of the metabolites were obtained with the use of an Agilent 1290 LC system connected to a hybrid Q-TOF tandem mass spectrometer (Agilent 6540 Series Accurate Mass Q-TOF-MS/MS). The chromatographic conditions of the LC-MS/MS analysis were identical to those in the metabolic study (see above, “LC-MS assay for t_0.5_ evaluation”). The Dual ESI source was operated in positive ion mode with the following conditions: the fragmentor voltage was set at 150V, nebulizer gas was set at 40 psig, capillary voltage was set at 3000 V, drying gas flow rate and temperature were set at 10 L/min and 300°C, respectively. The MS was operated in targeted MS/MS acquisition mode with a fixed collision energy of 35 V and a mass range of 30–1700 m/z. The ions that displayed m/z values, retention times and delta retention times specific for the compounds of interest were selected for fragmentation. The instrument was operated in High Resolution mode (4 GHz). The Q-TOF was calibrated using reference masses 121.050873, 149.02332 and 922.009798 during the analysis run to ensure constant mass correction.

## Results

### Half-life values evaluated using an *in vitro* approach

**Fig 1:**
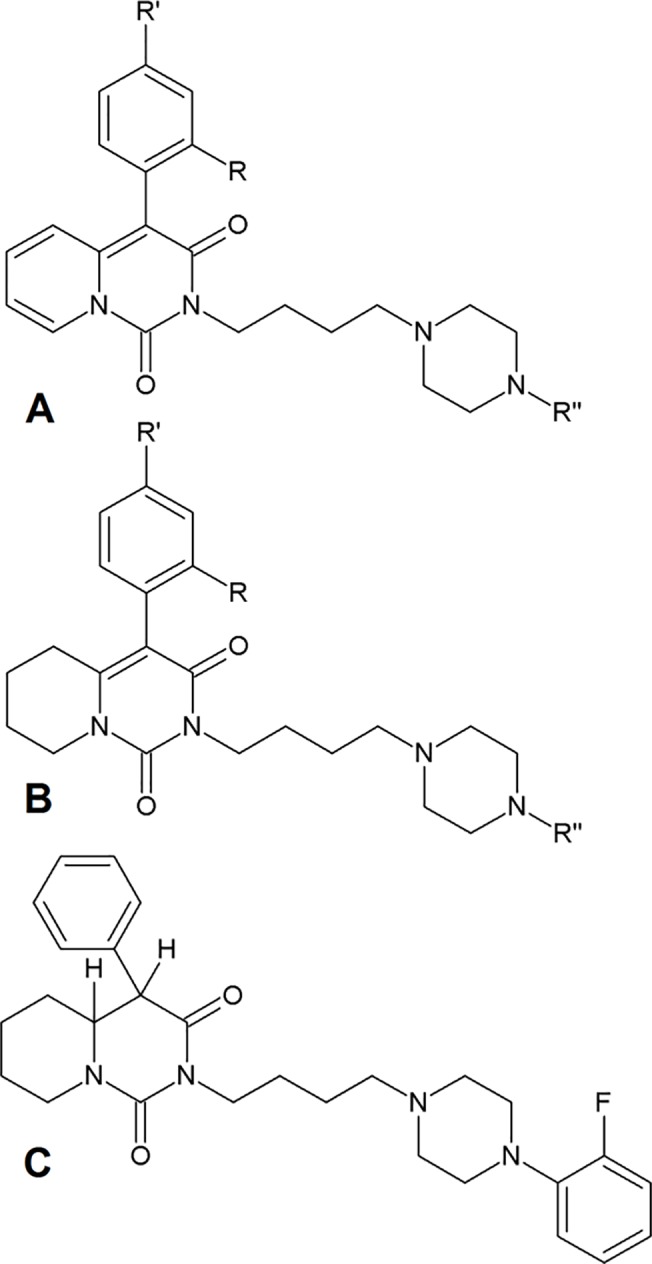
Structures of studied compounds. A) General structure of compounds 1–17 B) General structure of compounds 18–30 C) Structure of compound 25

**Fig 2:**
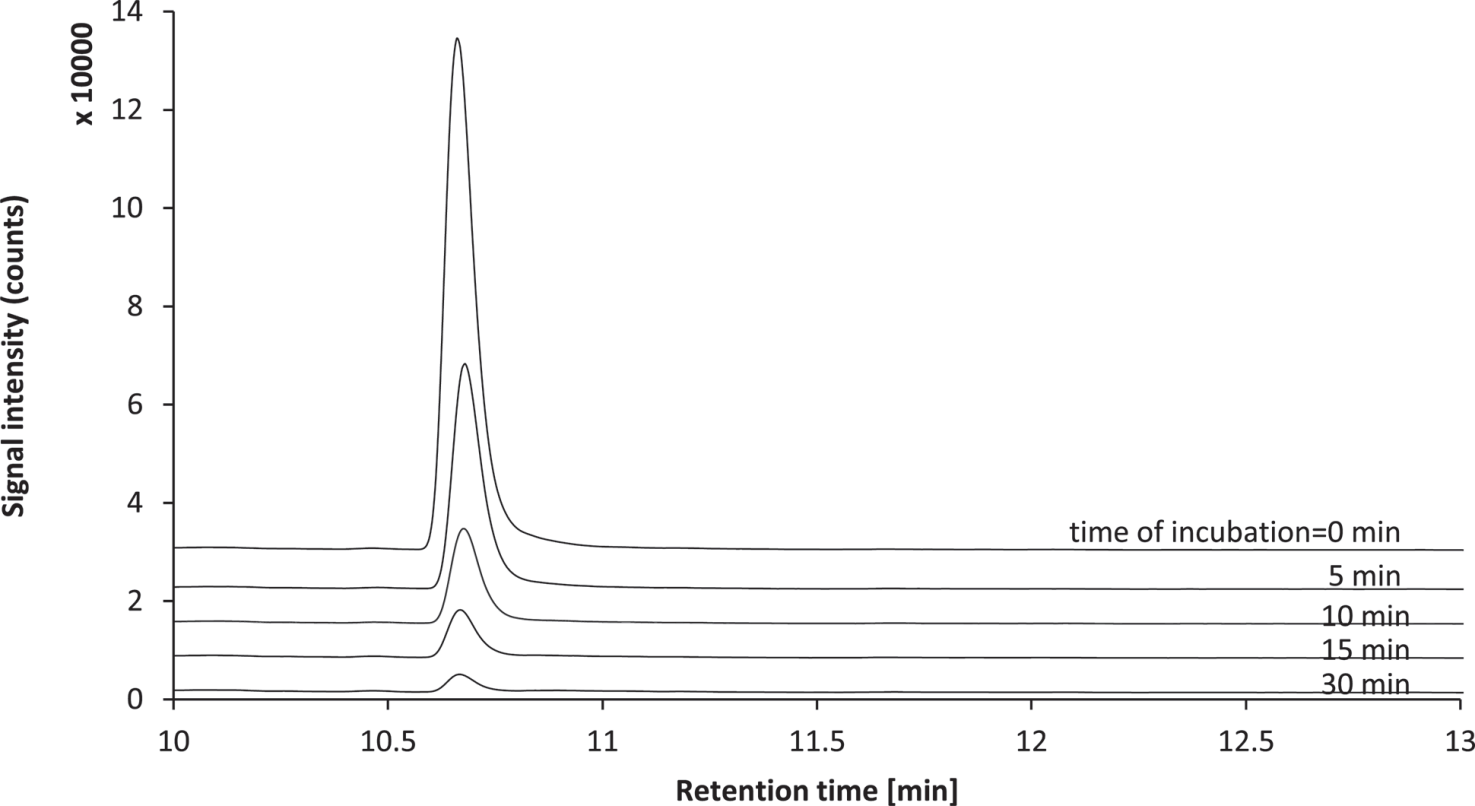
Time course of biotransformation of compound 3 in the presence of HLM and NADPH. Example single ion monitoring chromatograms (SIM, m/z = 503.22) obtained for samples taken immediately after addition of HLM and after 5, 10, 15 and 30 minutes are shown.

**Fig 3:**
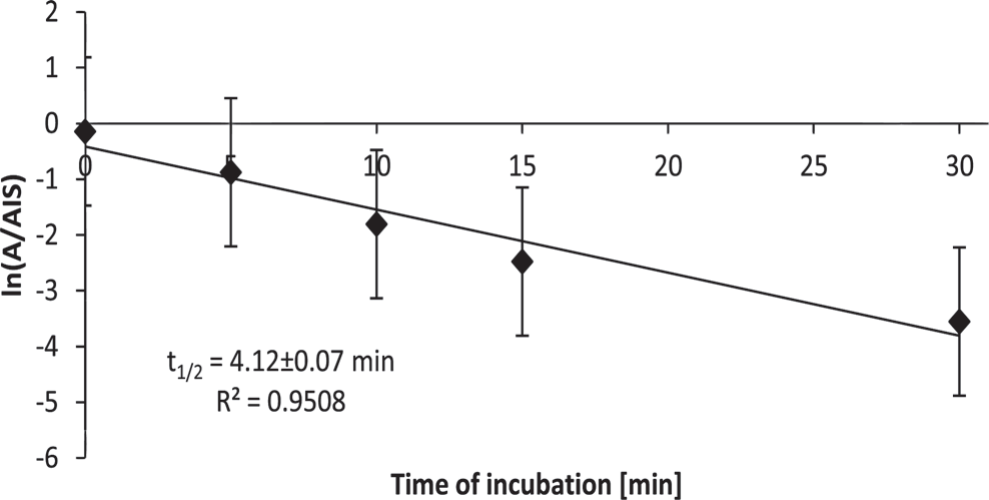
Plot showing relationship used to calculate t_0.5_. The average value of the natural logarithm of the compound peak area to internal standard peak area ratio are plotted.

The general structure of the studied arylpiperazine derivatives is shown on Fig 1. Table 1 presents average half-life values in minutes, for both studied derivatives and most popular arylpiperazine drug available on market—buspirone. After 30 minutes of incubation, all of the derivatives were metabolized, as evidenced by the parent compound peak areas for all compounds existing at less than 30% of the starting peak areas (Fig 2). As shown in Fig 3, after transforming the peak area/internal standard peak area ratio using a logarithmic function, a linear dependence was achieved, according to Equation 1. To evaluate the biotransformation half-life, only the linear part of the plot was used. The values obtained for t_0.5_ ranged from 3 to 9 minutes. No clear rules can be found regarding the relationship between particular substituents and the obtained half-life values.

### Half-life value prediction using SVM regression

**Fig 4:**
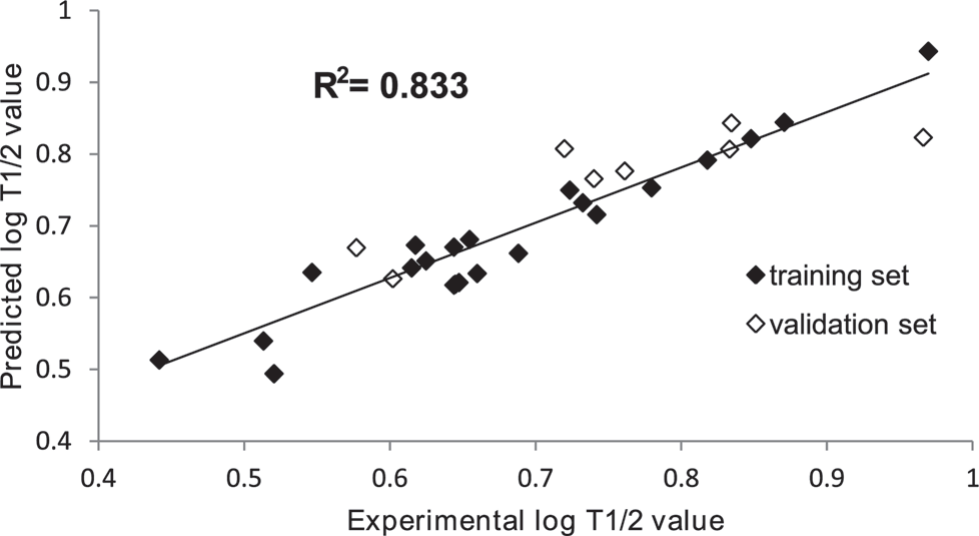
Correlation between the experimental log t_0.5_ values and the ones predicted by Support Vector Machine learning. The correlation coefficients are R’ = 0.939 for the training set, R” = 0.844 for the test set and R”’ = 0.913 for both sets. The mean squared error was MSE’ = 0.002 for the training set, MSE” = 0.005 for the test set and MSE”’ = 0.003 for both sets.

Descriptors used to create the SVM-based regression model were chosen with the data-mining variable selection feature of Statistica software. All of the molecular descriptors chosen by the variable selection method belonged to edge adjacency indices (namely: Eig11_EA(bo), Eig10_EA(bo), SpMax_AEA(ri), Eig01_AEA(ri), SpDiam_AEA(ri), SpMax_EA, detailed descriptions contained in the supplemental data) which are topological molecular descriptors. Briefly, they are derived from the edge adjacency matrix, which encodes the connectivity between molecular graph edges. After the successful creation of the SVM model, the predicted half-life values and observed half-life values were plotted against each other for both data sets (Fig 4) to visualize the accuracy of the model. The correlation coefficients were R’ = 0.9369 for the training set, R” = 0.8440 for the test set and R”’ = 0.9130 for both sets. The mean squared error was MSE’ = 0.002 for the training set, MSE” = 0.005 for the test set and MSE”’ = 0.003 for both sets.

### Determination of Biotransformation pathways: LC-Q-TOF-MS/MS assay for metabolite structural elucidation

Several derivatives that varied in their degrees of non-pharmacophore ring saturation, R, R’ substituents and pharmacophore parts (2-pyridyl, 2-pyrimidynyl, phenyl-CF_3_), were chosen to collect fragmentation spectra using a Q-TOF tandem mass spectrometer and LC conditions as described earlier. The acquired fragment mass spectra revealed that diverse biotransformation pathways acted upon these compounds. The compounds’ structures and chromatographs were submitted to MassHunter Workstation Software MetaboliteID ver. B.02.00 build 2.0.121 to detect possible biotransformation products. Based on a comparison between the fragmentation pathways of parent compounds and their metabolites, it was possible to determine the main types of transformations that occurred, as well as designate transformation-vulnerable sites among the studied molecules.

**Fig 5:**
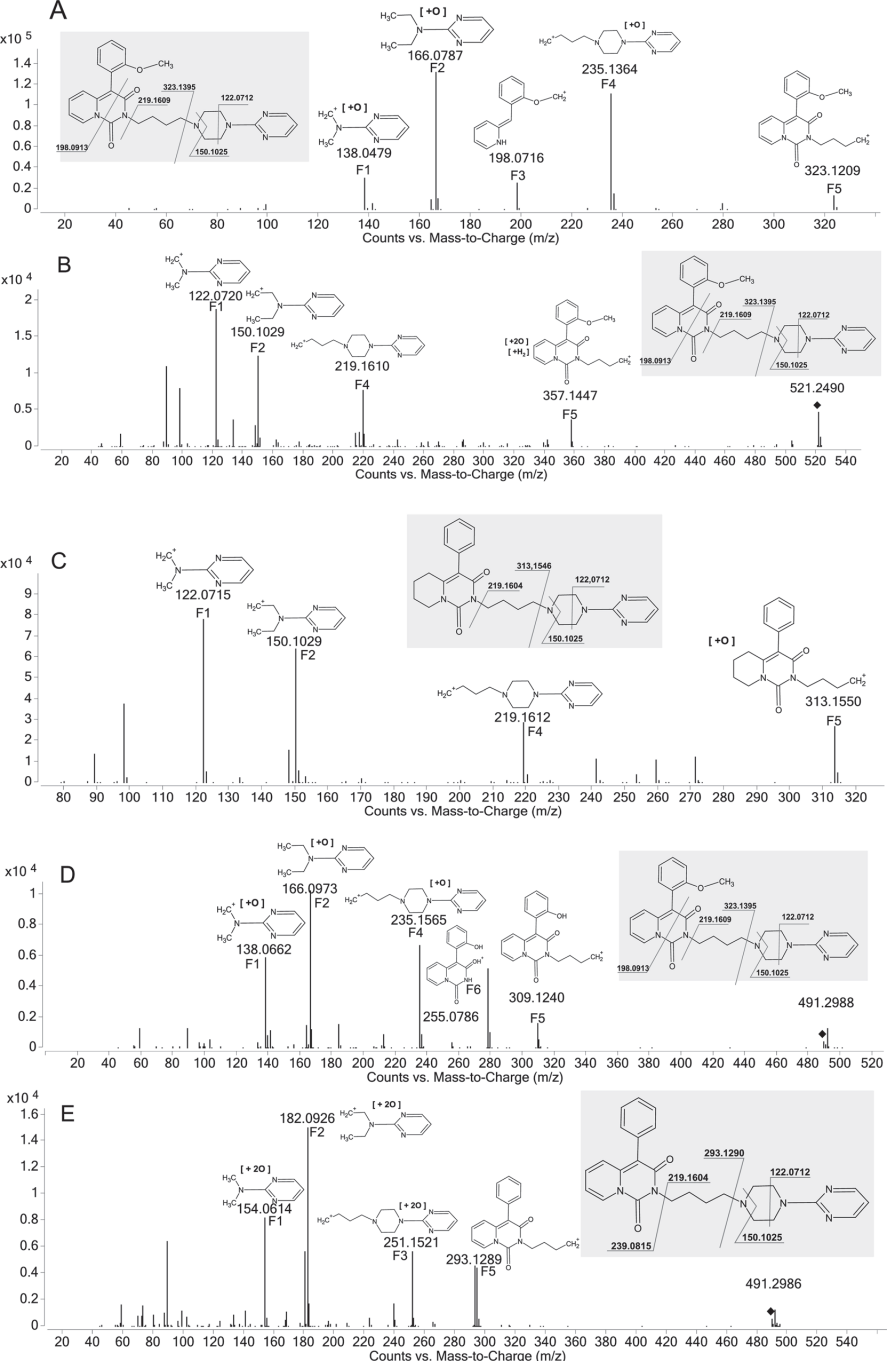
Q-TOF-MS/MS fragmentation spectra of protonated metabolites, along with fragmentation schemes of parent compounds indicated in grey boxes. A) Hydroxylation in the pharmacophore part; example shown is compound 11 metabolite M1. B) Water addition and reduction of the unsaturated ring in the non-pharmacophore part; example shown is compound 11 metabolite M4. C) Hydroxylation in the non-pharmacophore part of the molecule; example shown is compound 30 metabolite M2. D) O-demethylation and hydroxylation; example shown is compound 11 metabolite M3. E) Double hydroxylation in the pharmacophore part of the molecule; example shown is compound 6 metabolite M3.

The most common metabolic reaction, occurring in every studied compound, was a hydroxylation in the pharmacophore part of the molecule (Fig 5A). Analysis of the obtained spectra revealed fragments of the pharmacophore part with their masses increased by 16 Da, indicating oxidation. The second most common reaction among the studied compounds was the addition of water combined with reduction of the unsaturated ring in the non-pharmacophore parts of the molecules, resulting in a mass increase of 34 Da (Fig 5B). A less common metabolic pathway, namely hydroxylation of the non-pharmacophore part of the molecules, was observed on the M1 and M2 metabolites of compound 30 (Fig 5C). Fig 5D shows a pathway unique to compounds with the methoxyl moiety. Analysis of the spectrum for compound 11’s M3 metabolite revealed an O-demethylation reaction along with hydroxylation in the pharmacophore part. Metabolite M3 of compound 6 underwent a rare reaction, double hydroxylation of the non-pharmacophore site of the molecule (Fig 5E). A summary of the proposed metabolic pathways along with the pseudomolecular m/z ratios and LC retention times of the formed biotransformation products has been included in the supporting information (S1 Table).

## Discussion

The set of arylpiperazine derivatives studied here provides an opportunity for research into Structure-Activity Relationships (SAR). Comparison of the studied compounds (Fig 1) shows structural homology, with substituents in locations R, R’ and R” changing in a pattern that should permit, in certain situations, the generation of simple conclusions regarding structure-property relationships, if such relationships exist. These compounds are a very promising group of drug candidates, not only because of their activity and selectivity, but also due to their mechanism of action [3,20]. Given the fact that metabolic stability is often a limiting factor in the drug development pipeline [1], a more detailed study of these compounds’ metabolic stability was necessary. This work permits comparison of the studied arylpiperazine derivatives with buspirone in terms of stability, and allows for comparison of the observed biotransformation pathways with current knowledge. Although previous research into modeling arylpiperazine biological properties (activity, selectivity and lipophilicity) has proven its value [5–7,10,21], generally, computational methods have been used mostly to predict sites of metabolism, the structures of potential metabolites or interactions of molecules with metabolizing enzymes [22]. Therefore, an attempt to try to quantitatively model metabolic stability in analogy with QSAR studies is a clear next step. Metabolic half-life, as assessed in the presence of human liver microsomes and NADPH, was chosen as a readily available quantitative property of a compound that reflects its susceptibility to first-pass effect. Metabolic half-life is a widely accepted factor that influences the decision-making process in the drug development pipeline. It is of note that some attempts at developing metabolically protected derivatives have already been made [23], which only proves that a need exists to expand knowledge about the structure-metabolic stability relationships of arylpiperazines and to develop new approaches that help to interpret experimental data. The comparison of characteristic moieties between structures and their half-life values should provide an answer and guidelines on how to develop more metabolically stable derivatives. Such an approach can give valuable results, as shown clearly in the report of Tandon and co-workers[23]. In the case of the 30 new arylpiperazine derivatives analyzed in this study, compounds 30 and 17 are the most metabolically stable compounds, with their half-life values being 9.32±0.04 and 9.25±0.34 min, respectively. Simple visual comparison of their structures reveals that both of those compounds have a 2-pyrimidynyl ring in the pharmacophore part of the structure. Clearly this moiety alone cannot be treated as a clue for improving metabolic stability of these compounds, however, as the t_0.5_ values for other derivatives with this ring range from 2.76±0.54 min (compound 13) to the previously mentioned 9.32±0.04 min (compound 30). The half-life values of compounds with hydrogen in the R position range from 5.29±0.57 min for compound 6 to 9.25±0.34 min for compound 17, showing that comparing other moieties in these derivatives also does not provide any direct conclusions about the structure-metabolic stability relationship.

**Fig 6:**
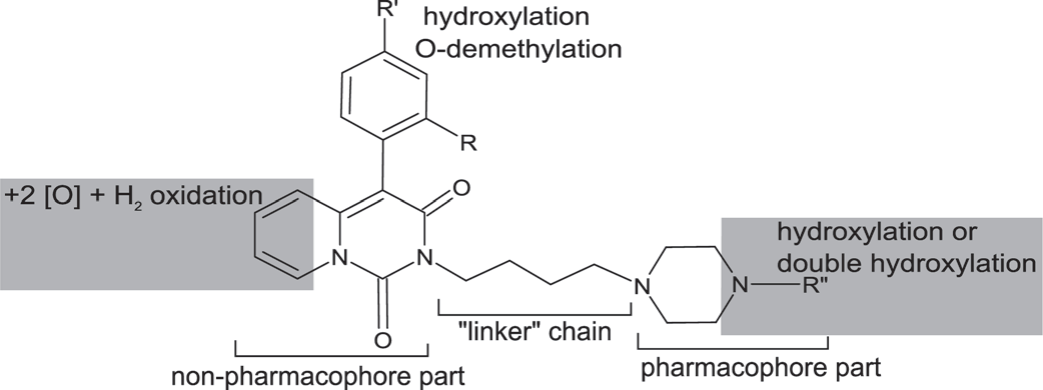
General structure of the studied compounds along with sites of metabolism and types of reactions that occur in those sites.

The analysis of Q-TOF-MS/MS spectra provided valuable data concerning the biotransformation pathways of the studied derivatives, and proved that the studied chemical scaffold can undergo multiple and diverse transformations, the complexity of which is further increased by the properties of different substituents. The comparison of the MS/MS fragmentation spectra of parent compounds and their biotransformation products allowed for identification of a transformation-vulnerable site, a location in the general structure where certain types of reactions take place. To clarify the MS/MS fragmentation mechanism, ions identified in the spectra were named analogically based on their relationships to the general structure (Fig 5). A summary of the metabolites, obtained with the help of the MassHunter Workstation Software MetaboliteID and inspection of MS/MS spectra, is included in the supplementary material, in S2 Table. A reaction characteristic of all studied compounds was a hydroxylation in the pharmacophore part of molecule (Fig 5A). This conclusion can be drawn based on analysis of the fragmentation spectra of the metabolites, in which the m/z ratio was increased by 16 Da compared to the parent compounds’ monoisotopic masses. Along with the shorter retention times of those metabolites, indicating that they are more hydrophilic than their predecessors as a result of hydroxylation, hydroxylation is the most likely reaction in this case. When comparing fragmentation ions (namely F1, F2 and F4, Fig 5A) of the hydroxylated metabolite M1 of compound 11, a determination of the hydroxylation site becomes possible. Although all three fragments have an increased m/z ratio value, the presence of all of them alongside the F3 and F5 ions not only excludes the “linker chain” as a possible hydroxylation site (because the mass of the F5 ion has not changed), but also leads one to believe that hydroxylation occurs in the site represented in Fig 6, which is a part of the piperazine ring and the R” moiety (the pharmacophore part). This finding is in agreement with a previously reported tendency for the hydroxylated pharmacophore to form metabolites, i.e., for buspirone [24]. Another reaction, leading to a change in mass of 34 Da, can be explained as an addition of water along with reduction of the unsaturated ring in the non-pharmacophore part, consisting of a 4-aryl-2H-pyrido[1,2-c]pyrimidine-1,3-dione system. The fragmentation spectrum of the metabolite M5 of compound 11 (Fig 5B) presents evidence to support such a reaction. The unchanged m/z values of ions F1, F2, and F4 direct attention to the non-pharmacophore site of the molecule. The mass of ion F5 is increased by 34 Da, when compared to the parent compound’s fragmentation ion. This leads one to propose that a biotransformation-type reaction is the cause. This reaction can be described as a water addition and a reduction of the unsaturated ring in the locations indicated on the molecule in Fig 6. Predicting a more detailed site of metabolism for this type of biotransformation on the non-pharmacophore part of the molecule is more challenging because it has not been observed in previous studies on arylpiperazine metabolism [12,25]. The resulting metabolites possess a saturated ring and possibly a hydroxyl/carbonyl moiety. This reaction can lead to a set of isomeric structures, with variations in the position and geometry of the-OH substituent. The exact structures of products of this type of reaction cannot be revealed by the methods used here and was not the aim of this study. This reaction was observed only in the compounds with an unsaturated ring in this part of the molecule, which helps to elucidate the site of this transformation.

A less-frequent type of reaction in this set of compounds, also a hydroxylation in the non-pharmacophore part, was observed in the M2 metabolite of the compound 30 spectrum (Fig 5C). Comparison of the F1, F2, and F4 ions (with their m/z ratios unchanged) again directs attention to the other side of the molecule as the site of this reaction. With the presence of the F5 ion and an ion with its mass increased by 16 Da, the site for the hydroxylation reaction can be determined to be the non-pharmacophore site of the molecule.

The metabolite M2 of compound 30 is an example of a small change in mass (increase of only 2 Da), yet a specific and interesting reaction. Comparing the masses of F1, F2 and F5 ions in its spectrum (Fig 5D) reveals a hydroxylation, as described earlier. Ions F5 and F6, however, change their m/z ratio by -14 Daltons, which is the m/z ratio for a CH_2_ moiety, and evidence that an O-demethylation reaction is taking place in this part of the molecule. A change in the monoisotopic mass of only 2 Da represents O-demethylation (which decreases the mass by 16 Da) and hydroxylation (which increases the mass by 18 Da) reactions occurring in the same molecule. The final type of biotransformation reaction observed here is a double hydroxylation in the pharmacophore part. This reaction is exclusive to metabolite M3 of compound 6. By consideration of the F1, F2 and F3 ions, which have increases in their m/z ratios of 32 Da (indicative of addition of 2 oxygen atoms), and the F4 ion, which has no change in its monoisotopic mass, the site for a double hydroxylation reaction can be determined to reside in the pharmacophore parts of the studied compounds (Fig 6). Comparing this fact with the current state of knowledge about arylpiperazine metabolism, the observed biotransformations are rather unique for this group of compounds. The previously observed [24,25] N-dealkylation products were not found among our studied derivatives at detectable levels. Structural comparison of the studied compounds and buspirone suggests that a bulkier non-pharmacophore group might impose steric hindrance that prevents the N-dealkylation reaction. N-oxidation products, which are characteristic for buspirone metabolism, are hard to distinguish from hydroxylation products, and hence further studies of this biotransformation type are needed. The fragmentation scheme of the studied arylpiperazines was found to be analogous to the one observed in the above-mentioned cited study.

With no clear structure-metabolic stability relationships observed, the development of a mathematical model to predict the half-life value from 3D structure descriptors needed a more sophisticated technique than simple linear methods. The Multiple Linear Regression analysis conducted here yielded results that were far from satisfactory, with the correlation coefficients R = 0.73 and R^2^ = 0.53 for the model created, thus proving a need to use other computational methods. Support Vector Machines, a non-linear regression technique, proved to be of great use for the present study. With a set of the 6 most significantly important descriptors (as chosen by the feature selection option in Statistica Software), SVM was able to develop a predictive model with correlation coefficients R = 0.8440 for test set and R = 0.9130 for both training and validation sets. The mean squared error was MSE = 0.005 for the test set and MSE = 0.003 for both sets. These results demonstrate the complex character of structure-metabolic stability relationships among arylpiperazines. The validation values also prove that the developed model is a valid and reliable tool for half-life value prediction for novel arylpiperazine derivatives. All of the molecular descriptors chosen by the feature selection method belonged to edge adjacency indices, which are topological molecular descriptors. They are derived from the edge adjacency matrix, which encodes the connectivity between graph edges, and are based mostly on the two-dimensional structure of a molecule.

Even though the chosen descriptors are based on a two-dimensional molecular graph, a comparison of descriptor values (two-dimensional model versus optimized three-dimensional model) showed a difference. The reason for the difference in values, which on first inspection should be equal, is the type of property by which the descriptors are weighted. In the model developed here, molecular descriptors based on a two-dimensional molecular graph are weighted by either the bond order or resonance integral, both of which are properties that differ in two-dimensional structures from three-dimensional optimized structures. This proves not only that the three-dimensional structure is significant for metabolic stability, but also that three-dimensional optimization is a necessary step for building a quantitative metabolic stability-structure relationship model.

As the search for more active or selective arylpiperazines continues, metabolic stability research should be another important factor to take under consideration when developing new derivatives. Experimental studies on small-molecule metabolism are resource-and time-consuming, hence the need for computational methods that complement *in vitro* approach. The presented results show that SVM regression is a valid tool for predicting metabolic stability using structural data as an input for the model. In this study, a simple and accurate procedure for evaluating half-life, combined with *in silico* calculations based on the SVM technique, and finalized by analysis of LC-MS/MS spectra provides a comprehensive and reliable tool for investigating metabolic stability and factors that affect it. It also reflects the complex nature of structure-metabolic stability relationships, which demand the use of advanced statistical techniques to create appropriate descriptive models. The proposed approach, combining DFT calculations, data-mining techniques and experimental data, provides a complete procedure to establish a model that, locally, in a given chemical space, is able to describe and quantitatively predict metabolic half-life *in vitro*. Taking into account a small size of the dataset, a fact needs to be highlighted that developed model is suitable only to predict the metabolic stability of arylpiperazine derivatives, that don’t differ much in chemical structure from compounds used to build the model. The proposed procedure, however, can be used for other groups of compounds, developing analogous models for predicting their metabolic stability in their unique, chemical space. A goal to develop an *in sillico* approach, that enables prediction of metabolic half-life for any compound of interest seems to be beyond current possibilities. First of all, the establishment of any empirical model is based on the approximation of model coefficients. This approximation would be better for restricted chemical space rather than for undefined and diversified chemical space of all drugs, drug candidates, and generally xenobiotic CYP’s substrates. The building of general model will require plenty of reliable experimental results (preferably obtained with the same procedure) and the choice of molecules used can be always questioned, because of insufficient representation of some chemical structures. Second important issue, that should be discussed is the complex origin of a final value of metabolic half-life. This parameter was chosen because of importance for real-life practical implementation and decision making process. The half-life value is a result of a susceptibility to and a rate of many simultaneously occurring metabolic reactions, that lead to the formation of a set of metabolites. In the proposed approach, the in-depth knowledge that lay beneath the observed half-life value is not necessary, but on the other hand the model’s output—predicted half-life can be directly utilized as a cut off in decision making process. Apart from providing an easy way to estimate metabolic stability, data on possible metabolic pathways and biotransformations of arylpiperazines are also supplied. The procedure can be easily applied for other chemical scaffolds, as well as adapted in different laboratories.

## Supporting Information

S1 Table Summary of biotransformation types among compounds chosen for Q-TOF analysis. Pseudomolecular ion mass is presented to compare m/z ratios of parent compounds and it's metabolites, allowing determination of possible biotransformation type. Structural specificity explains why the following compounds was chosen for Q-TOF analysis.
(XLS)

S2 Table Fragments structure and biotransformation types identification assay. All studied compounds were treated in a similar manner, but only compounds 11, 6 and 30 are used as examples to present an approach.
(DOC)
