# Supplementary material for: Prediction of Overall In Vitro Microsomal Stability of Drug Candidates Based on Molecular Modeling and Support Vector Machines. Case Study of Novel Arylpiperazines Derivatives
Source: PLoS One. 2015 Mar 31;10(3):e0122772. doi: 10.1371/journal.pone.0122772 (PMC4380424; doi:10.1371/journal.pone.0122772)
Supplement: S2 Table — All studied compounds were treated in a similar manner, but only compounds 11, 6 and 30 are used as examples to present an approach. (DOC) [file pone.0122772.s002.doc]

| **Compound 11  M=487.2452** | | | | | |
| --- | --- | --- | --- | --- | --- |
| Ion | Theoretical m/z | Measured m/z | Δppm | Molecular formula | Proposed structure |
| F1 | 323.1390 | 323.1212 | -55.0847 | C_19_H_19_N_2_O_3_ |  |
| F2 | 219.1604 | 219.1413 | -87.1508 | C_12_H_19_N_4_ |  |
| F3 | 122.0712 | 122.0538 | -142.54 | C_6_H_8_N_3_ |  |
| F4 | 150.1025 | 150.0840 | -123.249 | C_8_H_12_N_3_ |  |
| F5 | 198.0913 | 198.0719 | -97.9346 | C_13_H_12_NO |  |

**S2 Table. Fragments structure and biotransformation types identification assay.** All studied compounds were treated in a similar manner, but only compounds 11, 6 and 30 are used as examples to present an approach.

| **Compound 11 M1 M=503.2388 +O** | | | | | |
| --- | --- | --- | --- | --- | --- |
| Ion | Theoretical m/z | Measured m/z | Δppm | Molecular formula | Proposed structure |
| F1 | 138.0661 | 138.0479 | -131.821 | C_6_H_8_N_3_O |  |
| F2 | 166.0974 | 166.0787 | -112.585 | C_8_H_12_N_3_O |  |
| F3 | 198.0913 | 198.0716 | -99.4491 | C_13_H_12_NO |  |
| F4 | 235.1553 | 235.1364 | -80.3724 | C_12_H_19_N_4_O |  |
| F5 | 323.1390 | 323.1209 | -56.013 | C_19_H_19_N_2_O_3_ |  |

| **Compound 11 M2 M=503.2388 +O** | | | | | |
| --- | --- | --- | --- | --- | --- |
| Ion | Theoretical m/z | Measured m/z | Δppm | Molecular formula | Proposed structure |
| F1 | 122.0712 | 122.0518 | -158.9489 | C_6_H_8_N_3_ |  |
| F2 | 166.0974 | 166.0787 | -112.5972 | C_8_H_12_N_3_O |  |
| F3 | 198.0913 | 198.0716 | -99.4589 | C_13_H_12_NO |  |
| F5 | 323.1390 | 323.1209 | -56.0161 | C_19_H_19_N_2_O_3_ |  |

| **Compound 11 M3 M=489.2236 –CH_2_ +O** | | | | | |
| --- | --- | --- | --- | --- | --- |
| Ion | Theoretical m/z | Measured m/z | Δppm | Molecular formula | Proposed structure |
| F1 | 138.0661 | 138.0662 | 0.724291 | C_6_H_8_N_3_O |  |
| F2 | 166.0974 | 166.0973 | -0.60206 | C_8_H_12_N_3_O |  |
| F4 | 235.1553 | 235.1565 | 5.103011 | C_12_H_19_N_4_O |  |
| F5 | 309.1233 | 309.1240 | 2.264469 | C_18_H_17_N_2_O_3_ |  |
| F6 | 255.0764 | 255.0786 | 8.624867 | C_14_H_11_N_2_O_3_ |  |

| **Compound 11 M4 M=521.2507 +H2O2** | | | | | |
| --- | --- | --- | --- | --- | --- |
| Ion | Theoretical m/z | Measured m/z | Δppm | Molecular formula | Proposed structure |
| F1 | 122.0712 | 122.0720 | 6.553552 | C_6_H_8_N_3_ |  |
| F2 | 150.1025 | 150.1029 | 2.664846 | C_8_H_12_N_3_ |  |
| F4 | 219.1604 | 219.1610 | 2.737721 | C_12_H_19_N_4_ |  |
| F5 | 357.1444 | 357.1447 | 0.839996 | C_19_H_21_N_2_O_5_ |  |

| **Compound 11 M5 M=521.2507 +H2O2** | | | | | |
| --- | --- | --- | --- | --- | --- |
| Ion | Theoretical m/z | Measured m/z | Δppm | Molecular formula | Proposed structure |
| F1 | 122.0712 | 122.0714 | 1.638388 | C_6_H_8_N_3_ |  |
| F2 | 150.1025 | 150.1020 | -3.33106 | C_8_H_12_N_3_ |  |
| F4 | 219.1604 | 219.1597 | -3.19401 | C_12_H_19_N_4_ |  |
| F5 | 357.1444 | 357.1424 | -5.59998 | C_19_H_21_N_2_O_5_ |  |

| **Compound 30 M= 461.267** | | | | | |
| --- | --- | --- | --- | --- | --- |
| Ion | Theoretical m/z | Measured m/z | Δppm | Molecular formula | Proposed structure |
| F1 | 122.0712 | 122.0530 | -149.1155 | C_6_H_8_N_3_ |  |
| F2 | 150.1025 | 150.0837 | -125.2634 | C_8_H_12_N_3_ |  |
| F4 | 219.1604 | 219.1409 | -88.9838 | C_12_H_19_N_4_ |  |
| F5 | 297.1597 | 297.1405 | -64.6158 | C_18_H_21_NO_2_ |  |

| **Compound 30 M1 M=493.2568 +2O** | | | | | |
| --- | --- | --- | --- | --- | --- |
| Ion | Theoretical m/z | Measured m/z | Δppm | Molecular formula | Proposed structure |
| F1 | 122.0712 | 122.0360 | -288.4394 | C_6_H_8_N_3_ |  |
| F1’ | 138.0661 | 138.0286 | -271.6828 | C_6_H_8_N_3_O |  |
| F2 | 150.1025 | 150.0641 | -255.8906 | C_8_H_12_N_3_ |  |
| F2’ | 166.0974 | 166.0582 | -236.0618 | C_8_H_12_N_3_ |  |
| F4 | 219.1604 | 219.1409 | -88.9838 | C_12_H_19_N_4_ |  |
| F4’ | 235.1553 | 235.1153 | -288.4394 | C_12_H_19_N_4_O |  |
| F5 | 313.1546 | 313.1174 | -271.6828 | C_18_H_21_NO_3_ |  |
| F6 | 243.1128 | 243.0930 | -255.8906 | C_14_H_15_N_2_O_2_ |  |

| **Compound 30 M2 M=477.2615 +O** | | | | | |
| --- | --- | --- | --- | --- | --- |
| Ion | Theoretical m/z | Measured m/z | Δppm | Molecular formula | Proposed structure |
| F1 | 122.0712 | 122.0715 | 2.4575 | C_6_H_8_N_3_ |  |
| F2 | 150.1025 | 150.1029 | 2.6648 | C_8_H_12_N_3_ |  |
| F4 | 219.1604 | 219.1612 | 3.6502 | C_12_H_19_N_4_ |  |
| F5 | 313.1546 | 313.1550 | 1.2773 | C_18_H_21_NO_3_ |  |

| **Compound 30 M3 M=477.2615 +O** | | | | | |
| --- | --- | --- | --- | --- | --- |
| Ion | Theoretical m/z | Measured m/z | Δppm | Molecular formula | Proposed structure |
| F1 | 138.0661 | 138.0286 | -271.6828 | C_6_H_8_N_3_O |  |
| F2 | 166.0974 | 166.0590 | -231.2431 | C_8_H_12_N_3_ |  |
| F4 | 235.1553 | 235.1162 | -166.3007 | C_12_H_19_N_4_O |  |
| F5 | 297.1597 | 297.1213 | -129.2401 | C_18_H_21_NO_2_ |  |
| F5’ | 313.1546 | 313.1174 | -118.8052 | C_18_H_21_NO_3_ |  |
| F6 | 243.1128 | 243.0375 | -309.8287 | C_14_H_15_N_2_O_2_ |  |

| **Compound 6 M=457.2348** | | | | | |
| --- | --- | --- | --- | --- | --- |
| Ion | Theoretical m/z | Measured m/z | Δppm | Molecular formula | Proposed structure |
| F1 | 122.0712 | 122.0530 | -149.1155 | C_6_H_8_N_3_ |  |
| F2 | 150.1025 | 150.0837 | -125.2634 | C_8_H_12_N_3_ |  |
| F4 | 219.1604 | 219.1409 | -88.9838 | C_12_H_19_N_4_ |  |
| F5 | 293.1284 | 293.1064 | -75.0580 | C_18_H_17_N_2_O_2_ |  |
| F6 | 239.0815 | 239.0608 | -86.5888 | C_14_H_11_N_2_O_2_ |  |

| **Compound 6 M1 M=473.1975 +O** | | | | | |
| --- | --- | --- | --- | --- | --- |
| Ion | Theoretical m/z | Measured m/z | Δppm | Molecular formula | Proposed structure |
| F1 | 138.0661 | 138.0290 | -268.712 | C_6_H_8_N_3_O |  |
| F2 | 166.0974 | 166.0592 | -229.986 | C_8_H_12_N_3_O |  |
| F3 | 235.1553 | 235.1162 | -166.273 | C_12_H_19_N_4_O |  |

| **Compound 6 M2 M=489.2230 +2O** | | | | | |
| --- | --- | --- | --- | --- | --- |
| Ion | Theoretical m/z | Measured m/z | Δppm | Molecular formula | Proposed structure |
| F1 | 154.0611 | 154.0614 | 1.947279 | C_6_H_8_N_3_O_2_ |  |
| F2 | 182.0924 | 182.0926 | 1.098343 | C_8_H_12_N_3_ |  |
| F4 | 251.1502 | 251.1521 | 7.565194 | C_12_H_19_N_4_ |  |
| F5 | 293.1284 | 293.1289 | 1.705737 | C_18_H_17_N_2_O_2_ |  |

| **Compound 6 M3 M=489.2230 +O** | | | | | |
| --- | --- | --- | --- | --- | --- |
| Ion | Theoretical m/z | Measured m/z | Δppm | Molecular formula | Proposed structure |
| F1 | 138.0661 | 138.0472 | -136.891 | C_6_H_8_N_3_O_2_ |  |
| F5 | 293.1290 | 293.1085 | -69.9351 | C_18_H_17_N_2_O_2_ |  |
| F6 | 239.0815 | 239.0611 | -85.3266 | C_14_H_11_N_2_O_2_ |  |

| **Compound 6 M4 M=491.2398 +2O + 2H** | | | | | |
| --- | --- | --- | --- | --- | --- |
| Ion | Theoretical m/z | Measured m/z | Δppm | Molecular formula | Proposed structure |
| F1 | 122.0712 | 122.0708 | -3.27678 | C_6_H_8_N_3_ |  |
| F2 | 150.1025 | 150.1018 | -4.66348 | C_8_H_12_N_3_ |  |
| F4 | 219.1604 | 219.1585 | -8.66945 | C_12_H_19_N_4_ |  |
| F5 | 327.1339 | 327.1313 | -7.94782 | C_18_H_17_N_2_O_2_ |  |
| F6 | 273.0862 | 273.0869 | 2.563293 | C_14_H_11_N_2_O_2_ |  |
